# Postnatal Steroids in Preterm Infants: A Narrative Review Series—Part 1: Inflammatory Modulation and Respiratory Impacts

**DOI:** 10.3390/children13030384

**Published:** 2026-03-09

**Authors:** Phoenix Plessas-Azurduy, Anie Lapointe, Punnanee Wutthigate, Sarah Spénard, Marc Beltempo, Wissam Shalish, Guilherme Sant’Anna, Gabriel Altit

**Affiliations:** 1 Division of Clinical & Translational Research, Faculty of Medicine and Health Sciences, McGill University, Montreal Children’s Hospital, Montréal, QC H4A 3H9, Canada; phoenix.plessas-azurduy@mail.mcgill.ca; 2 Division of Neonatology, Department of Pediatrics, CHU Sainte-Justine, Université de Montréal, Montréal, QC H3T 1C5, Canada; anie.lapointe@umontreal.ca; 3 Department of Pediatrics, Faculty of Medicine Siriraj Hospital, Mahidol University, Bangkok 10700, Thailand; punnanee.wut@mahidol.edu; 4 Division of Neonatology, Department of Pediatrics, Montreal Children’s Hospital, Montréal, QC H4A 3H9, Canada; sarah.spenard.med@ssss.gouv.qc.ca (S.S.); marc.beltempo@mcgill.ca (M.B.); wissam.shalish@mcgill.ca (W.S.); guilherme.santanna@mcgill.ca (G.S.)

**Keywords:** extremely preterm infants, bronchopulmonary dysplasia, postnatal corticosteroids, inflammation, respiratory outcomes, individualized therapy, steroid dosing strategies, pulmonary development, monitoring tools, physiology-driven care

## Abstract

**Highlights:**

**What are the main findings?**

- Extremely preterm infants experience persistent pulmonary inflammation and impaired lung development, and postnatal corticosteroids can reduce inflammation, facilitate mechanical ventilation weaning, and improve short-term respiratory outcomes.
- Evidence from clinical trials shows meaningful differences between corticosteroid regimens, such as low-dose dexamethasone versus hydrocortisone, in efficacy and side-effect profiles, while emerging physiology-based monitoring tools may help individualize treatment.

**What are the implications of the main findings?**

- A more individualized, physiology-informed approach to postnatal corticosteroid therapy may optimize timing, dosing, and patient selection, improving respiratory outcomes while minimizing systemic risks.
- Integrating mechanistic insights with clinical evidence supports moving away from uniform protocols toward tailored strategies that balance therapeutic benefits with potential long-term trade-offs.

**Abstract:**

Extremely preterm infants often require prolonged respiratory support due to lung immaturity and inflammation, placing them at high risk of lung injury and development of bronchopulmonary dysplasia (BPD). In many of these infants, systemic postnatal corticosteroids are used to reduce lung inflammation, facilitate mechanical ventilation (MV) weaning and extubation, and improve short-term pulmonary outcomes. However, despite decades of clinical use, substantial variation persists in timing, choice of agent and dosing. These inconsistencies reflect a lack of strong evidence and a limited understanding of the systemic and organ-specific effects of therapy for a highly heterogenous population usually exposed to this medication. This narrative review addresses these gaps by integrating current knowledge of the inflammatory and respiratory effects of postnatal corticosteroids in extremely preterm infants. We explore how corticosteroids modulate pulmonary inflammation, their effects on lung development, and how they affect key clinical outcomes such as extubation success and BPD severity. We also examine evolving approaches to corticosteroid administration and dosing, highlighting the importance of individualized strategies informed by developmental and disease-specific considerations. Comparative data from randomized controlled trials are reviewed, including the efficacy and side-effect profiles of commonly used regimens. Current evidence supports judicious use of late low-dose dexamethasone, while early prophylaxis with inhaled or intratracheal steroids remains experimental and is not routinely advised. In line with a physiology-driven approach, we also discuss emerging domain-specific monitoring tools that may enhance patient selection and optimize timing of intervention. By synthesizing mechanistic insights with clinical evidence, this review supports a more nuanced, individualized approach to postnatal corticosteroid therapy in extremely preterm infants, balancing therapeutic benefits with potential systemic trade-offs.

## 1. Introduction

The use of systemic postnatal corticosteroids in extremely preterm infants has been controversial for decades [1,2,3]. While these agents are widely administered after the first postnatal week to facilitate MV weaning and extubation and to mitigate the progression of lung injury, their broader physiologic effects are less well understood [4,5,6]. In evaluating these effects, it is important to distinguish more historical steroid use aimed at preventing BPD by dampening initial inflammatory triggers from the more recent shift toward treating evolving or established disease, where the focus is on reducing ongoing inflammation to facilitate liberation from mechanical support. Not surprisingly, despite their long-standing role in neonatal care, there is no standardized approach to time of initiation, dosing, or monitoring, leading to significant variations in practice across institutions [7,8,9]. Postnatal corticosteroids have been shown to significantly reduce pulmonary inflammation; however, their precise effects on the developing pulmonary system of the preterm neonate, such as impacts on lung water content and lung aeration, have yet to be fully elucidated. More recently, advances in physiological monitoring and imaging modalities have offered new avenues for evaluating pulmonary status that can potentially help refine how and when corticosteroids are used for neonatal pulmonary management. This review examines the effects of systemic postnatal corticosteroid use on the respiratory system and highlights emerging tools that may enable research avenues for more individualized and developmentally informed approaches in preterm infants. As the first installment of this narrative review series, this part focuses specifically on inflammatory modulation and its impacts on the respiratory system, while subsequent sections will address systemic and neurodevelopmental outcomes.

## 2. Prematurity and Evolving Lung Injury

The exposure of vulnerable premature lungs to intra- and extra-uterine insults results in pulmonary inflammation, fibrosis, alveolar simplification, parenchymal injury, airway and vascular remodeling, as well as impaired lung development [2]. In 1967, Northway described a lung disease in preterm infants called bronchopulmonary dysplasia (BPD) characterized by airway injury, inflammation, and fibrosis in relatively mature preterm infants exposed to high oxygen and MV [10]. Many decades later, a “new BPD” has been noted in more immature newborns treated with surfactant. This new BPD is characterized by disrupted lung development, marked by alveolar simplification and impaired vascular growth [11]. Moreover, the incidence of this new BPD is inversely proportional to the gestational age (GA) at birth and directly related to the use and duration of MV and oxygen supplementation [12]. This underscores how BPD risk factors represent a multifactorial continuum of intra- and extra-uterine insults, where prenatal stressors, including complex maternal conditions, transition into postnatal challenges such as infection, nutritional management, and the cumulative inflammatory injury driven by mechanical ventilation and oxygen toxicity. In extremely premature newborns (born at ≤28 weeks GA), BPD is often therapeutically defined and classified as mild/grade 1, moderate/grade 2, or severe/grade 3, based on respiratory and/or oxygen support exposure at 36 weeks post-menstrual age (PMA) [13]. Various definitions are shown in Table 1 [11,13,14,15,16,17]. Although the definitions remain controversial, most have shown associations between severe BPD with decreased long-term pulmonary function and neurodevelopmental outcomes, as demonstrated in longitudinal studies [18,19,20,21]. BPD remains challenging to define, and the overall clinical picture is often underrecognized, which may lead to an underestimation of disease severity. Historically, BPD definitions were primarily oxygen-based, relying on the duration and concentration of supplemental oxygen (FiO_2_) to determine disease presence and severity. However, these definitions are often confounded by institutional variations in oxygen saturation targets and titration practices. In contrast, support-based definitions, which classify BPD based on the level of respiratory support (e.g., mechanical ventilation, CPAP, or high-flow nasal cannula), have emerged with the aim of providing a more objective assessment. The Jensen classification [16] has gained popularity in clinical research since it was developed using an evidence-based approach, regardless of FiO_2_ requirements, that is said to better predict long-term morbidities, such as childhood neurodevelopmental impairment and late respiratory death, compared to traditional oxygen-based criteria. However, these support-based definitions are increasingly constrained by the expanding array of non-invasive respiratory modalities, which introduce substantial variability across centers and clinicians in how respiratory support is applied. The heterogeneity of these definitions as well as between-center variability significantly impact the interpretation of postnatal corticosteroid trials. Since BPD is “therapeutically defined,” the criteria used to enroll patients or define primary outcomes often reflect the era and the specific institutional protocols in place at the time of the study. This lack of a universal gold standard makes it challenging to compare the efficacy of steroid regimens across different trials, as an infant classified with “severe BPD” under one definition might only meet “moderate” criteria under another. This variability underscores the need for a shift toward physiology-driven markers to complement these support-based definitions in future research.

Rates of moderate to severe BPD among infants born at ≤28 weeks’ gestation range between 30 and 60% and have been stable or have even increased over the past few decades according to large neonatal databases [24,25]. The NICHD Neonatal Research Network reported that rates of BPD have remained similar between 1995 and 1996, 1997 and 2002, and 2003 and 2007 [26,27], and a report including 355,806 infants born between 2000 and 2009 from 669 North American hospitals in the Vermont Oxford Network reported that BPD rates varied from 26.2% to 30.4% without clinically relevant decline during that period [28]. A Swedish population-based study comparing two national cohorts of infants born at 22–26 weeks’ gestation between 2004 and 2007 and 2014 and 2016 found that despite increased survival and longer durations of both invasive and non-invasive respiratory support, the overall incidence of BPD remained unchanged, while severe BPD modestly declined among survivors at 36 weeks PMA [29]. Experts suggest that this trend may be driven by several factors, including the survival of more fragile and immature infants, expanding the limits of viability, adoption of higher oxygen saturation targets that prolong oxygen dependence, more complex maternal conditions, an increase in medically assisted pregnancies, and greater survival of twins or triplets due to advances in antenatal care [30]. BPD is fundamentally a disorder of impaired lung development, characterized by alveolar simplification and fibrosis [2]. Since definitions are based on therapeutic exposure, the criteria for diagnosing and grading BPD are heavily influenced by unit-specific protocols and clinical practices. Unit-specific and practitioner-dependent factors such as saturation targets, apnea thresholds and ventilation strategies contribute to variability in diagnosis. Moreover, defining BPD solely based on the need for oxygen or respiratory support at 36 weeks PMA overlooks the complex and heterogeneous involvement of the disease across the parenchymal, vascular, and airway compartments. BPD also involves pulmonary vascular disease, affecting arteries, veins, capillaries, and likely lymphatics, as supported by histopathological and imaging studies documenting vascular remodeling and lymphatic involvement in severe BPD cases [31,32].

**Figure 1:**
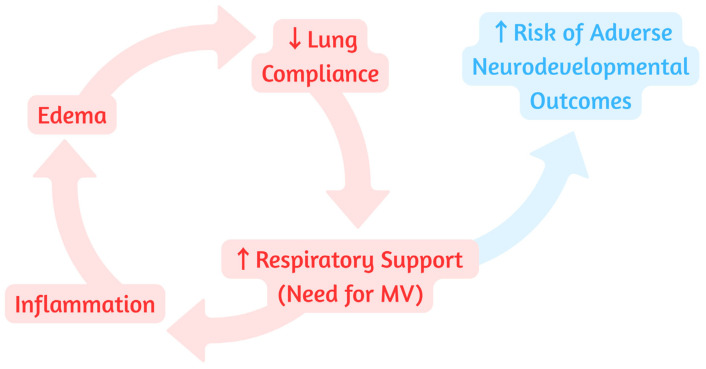
The vicious cycle of inflammatory lung injury and the neurorespiratory link. Abbreviations: MV (mechanical ventilation).

**Figure 2:**
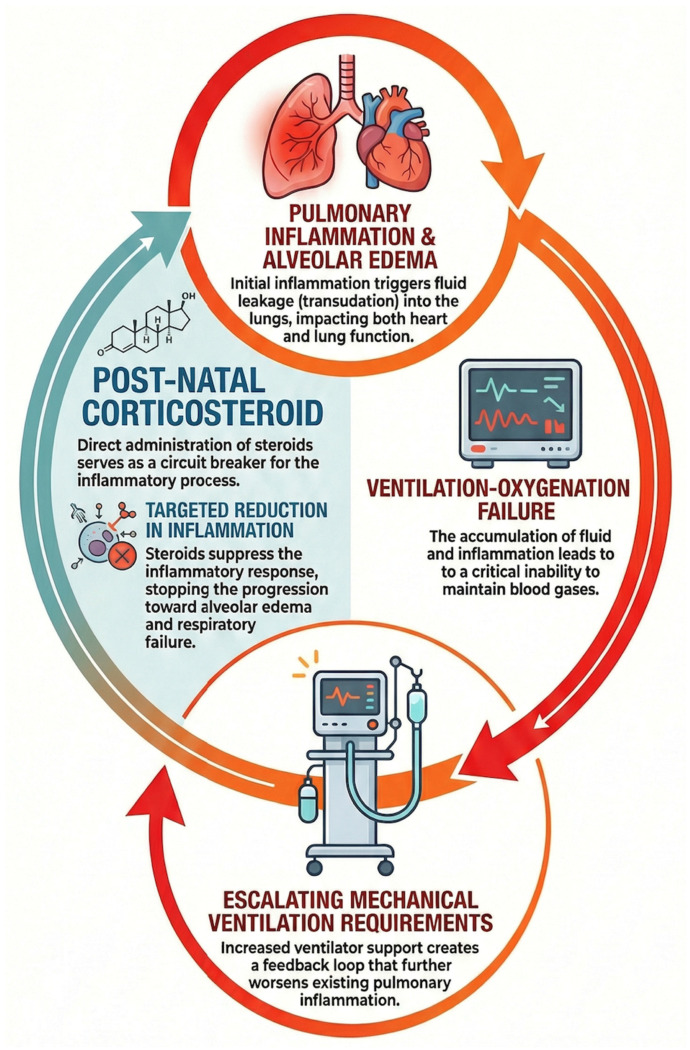
Postnatal corticosteroids, inflammatory lung disease and the preterm heart.

In addition, BPD is associated with significant pulmonary inflammation, which may lead to excessive transudation within pulmonary alveoli [2]. This inflammation leads to ventilation and oxygenation failure, warranting an increase in MV support [33]. Exposure to increasing ventilatory pressures and higher oxygen concentrations can exacerbate pulmonary inflammation, creating a self-perpetuating inflammatory cascade (Figure 1) that hinders lung healing and repair [34]. Systemic postnatal corticosteroids act as potent anti-inflammatory agents potentially interrupting this process to allow for an improvement in pulmonary compliance (Figure 2) [35]. Decreasing this inflammation may also allow for a reduction in alveolar transudation and edema, improving ventilation–perfusion mismatch [36]. As such, in extremely preterm infants, evolving lung disease reflects pulmonary inflammation and a broader systemic inflammatory cascade that can impair the development of other vulnerable organs, such as the brain and cardiovascular system, during a critical window of maturation.

BPD is also compounded by altered breathing control and neuromuscular dysfunction, which likely impact diaphragmatic and accessory respiratory muscle function [37]. Intermittent desaturations and episodes of hypoxia in premature infants with evolving lung disease are a concern for the developing brain as well [38]. Despite variability in its applied definition, a study compared different BPD classifications in a cohort of 218 survivors at 7–8 years old [39], including the 2021 Jobe definition [13], the 2018 NICHD definition [14], and the Victorian Infant Collaborative Study (VICS) 2005 definition, which was adapted from the 2019 Jensen classification [16]. The study found that, compared to infants with no BPD, those receiving oxygen up to 29% but without positive pressure support at 36 weeks PMA had an increased risk of abnormal respiratory function but no significant impact on neurodevelopmental outcomes [39]. In contrast, infants requiring ≥30% oxygen or any positive pressure support at 36 weeks PMA had a higher risk of both abnormal respiratory function and adverse neurodevelopmental outcomes. While infants requiring higher levels of respiratory support at 36 weeks PMA have a higher risk of adverse neurodevelopmental outcomes, it is important to clarify that this strong association between respiratory severity and neurodevelopment does not imply direct causation. The developmental trajectory of the brain in these infants is influenced by a multifactorial continuum of insults.

Systemic postnatal corticosteroids have been investigated as a strategy to reduce pulmonary inflammation and promote lung recovery. While historically linked solely to facilitation of extubation or “prevention” of BPD, this framing oversimplifies their current role. Over time, with the emergence of “new BPD” and increasingly immature babies, the primary therapeutic goal of postnatal steroid administration has now shifted to improve survival and blunt the ongoing inflammatory cascade within the immature lung, which if left unchecked can contribute to progressive lung and brain injury, impaired alveolar development, and systemic consequences, including death. Therefore, even when extubation is not immediately achieved, judicious use of corticosteroids may decrease mortality, attenuate inflammation, reduce ventilator dependence over time, and prevent the development of chronic fibrotic changes that limit lung growth. Therefore, creating individualized care plans for infants with evolving lung disease who are most likely to benefit from this intervention is essential to balancing potential benefits with known short- and long-term risks. Ultimately, the current aim of postnatal steroid administration is to interrupt this ongoing vicious cycle of lung and systemic inflammation, poor lung compliance, and escalating ventilatory support that can ultimately culminate in high morbidity and mortality.

## 3. Postnatal Corticosteroids

### 3.1. Anti-Inflammatory Mechanisms of Action

Glucocorticoids (GCs) and mineralocorticoids (MCs) are the two classes of corticosteroids that differ in their primary physiologic roles: GCs mainly regulate inflammation and immune responses, while MCs primarily impact electrolyte and fluid balance (namely, by retaining salt and water and excreting potassium) [40,41]. However, it should be noted that natural steroids possess both GC and MC activity to some extent [42].

Glucocorticoids are rapidly absorbed and generally have good oral bioavailability, typically ranging from 60% to nearly complete absorption [43]. In the body, more than 90% of endogenous cortisol circulates bound reversibly to proteins such as albumin and transcortin (also called corticosteroid-binding globulin) [44]. Synthetic corticosteroids bind in a similar way, although their degree of protein binding differs depending on the specific agent [45]. The unbound fraction represents the pharmacologically active form that crosses cell membranes to exert molecular effects. At higher circulating concentrations, corticosteroid-binding globulin becomes saturated, increasing the proportion of free steroid available to tissues. Importantly, premature and critically ill infants have lower corticosteroid-binding globulin levels, which raises the amount of unbound steroid. Because total serum cortisol assays measure both bound and free hormones, they can underestimate adrenal insufficiency and lead to unnecessary replacement therapy in this population [43].

Glucocorticoids are metabolized in the liver through phase I oxidation and reduction reactions, followed by phase II conjugation with inactive metabolites eliminated by the kidneys [44]. In the circulation, steroids are bound to transcortin and albumin, but the unbound fraction represents the pharmacologically active form. These molecular actions lead to a reduction in alveolar transudation and edema, which improves ventilation–perfusion mismatch and enhances pulmonary compliance, facilitating weaning from mechanical ventilation. Given the immaturity of hepatic and renal systems in preterm infants, further studies are needed to better characterize the pharmacokinetics of systemic glucocorticoids used for prevention and treatment of lung injury in this group.

Endogenous steroids exhibit varying degrees of both glucocorticoid and mineralocorticoid activity [42]. Cortisol, synthesized from cholesterol in the adrenal cortex, is the main glucocorticoid produced by the body [43]. In contrast, aldosterone, primarily generated in the zona glomerulosa of the adrenal gland, serves as the principal mineralocorticoid, with its release controlled by the renin–angiotensin system [45]. Synthetic corticosteroid analogues are designed to selectively enhance glucocorticoid or mineralocorticoid activity and improve potency compared to natural hormones. The primary corticosteroids administered for their anti-inflammatory effects in infants with BPD include hydrocortisone, dexamethasone, prednisolone and methylprednisolone, in addition to budesonide and betamethasone. A summary of each of these steroids’ pharmacological properties and relative potencies can be found in Table 2. The evidence of use in preterm infants with evolving BPD, primary clinical role and level of evidence for each steroid and dosing regimen is provided in Table 3.

#### 3.1.1. Hydrocortisone

Hydrocortisone is a corticosteroid frequently administered to manage hypotension and, in certain cases, to treat refractory pulmonary hypertension in neonates [66]. Adrenal replacement dosing focuses on physiological substitution, while anti-inflammatory dosing involves supraphysiologic levels necessary to treat BPD. Early low-dose hydrocortisone (prevention) must be distinguished from late high-dose regimens (treatment). The glucocorticoid and mineralocorticoid activities of commonly used corticosteroids are typically described relative to hydrocortisone, the synthetic analog of endogenous cortisol [67]. Hydrocortisone primarily exerts glucocorticoid effects but retains some mineralocorticoid activity, especially at supraphysiologic doses [40,68]. These higher doses are often required to modulate the dysregulated inflammatory response underlying lung injury in preterm infants. Due to its rapid oral absorption and short half-life, hydrocortisone is also well suited for cortisol replacement in cases of hypothalamic–pituitary–adrenal (HPA) axis suppression, which may occur following prolonged glucocorticoid exposure [43,68]. It should be noted that, while generally well-tolerated, hydrocortisone has also been associated with an increased risk of spontaneous intestinal perforation, particularly when used early in life or in combination with NSAIDs [69].

#### 3.1.2. Dexamethasone and Betamethasone

Dexamethasone is a long-acting, highly potent glucocorticoid, approximately 25 times more potent than hydrocortisone, with no mineralocorticoid activity [40]. It binds primarily to albumin (not transcortin), with about 75% of the drug protein-bound in plasma. Unlike prednisolone and other glucocorticoids, dexamethasone is not metabolized by 11β-hydroxysteroid dehydrogenase (type 1 or 2), does not require hepatic activation, and directly binds to glucocorticoid receptors to exert its effects [70]. This direct receptor binding drives potent inhibition of pro-inflammatory gene transcription and suppression of cytokine production in the developing lung, mechanisms thought to contribute to its anti-inflammatory efficacy. Early high-dose regimens have been linked to impaired cerebellar development and subcortical grey matter volumes, likely due to cell growth arrest during critical maturation windows [71].

Betamethasone is an isomer of dexamethasone differing only in the orientation of one methyl group exhibiting immunosuppressive and anti-inflammatory properties in a similar mechanism to that described above which has a longer half-life and a slower onset [72]. It has mostly been described for its use antenatally for women at risk of preterm delivery [73].

#### 3.1.3. Prednisone/Prednisolone and Methylprednisolone

Prednisolone and methylprednisolone are intermediate-acting synthetic glucocorticoids with comparable potencies and are often used interchangeably [74]. Prednisone, a prodrug, is converted to prednisolone by 11β-HSD1 in the liver [43]. Methylprednisolone, which has similar glucocorticoid activity, is preferred when intravenous administration is required since it does not require hepatic activation. Like other systemic corticosteroids, both agents exert their anti-inflammatory effects by downregulating pro-inflammatory cytokines and reducing leukocyte infiltration in lung tissue. These corticosteroids have been safely and effectively used in pediatric respiratory conditions, particularly asthma, and have an established safety profile in that context [58,75,76]. However, it must be emphasized that unlike dexamethasone, for which high-quality evidence from randomized trials exists, the use of prednisolone and methylprednisolone in BPD remains primarily supported by observational data and is largely focused on late-use scenarios (typically after 36 weeks PMA); consequently, they should not be considered therapeutic equivalents to dexamethasone at this time due to the lack of comparative randomized controlled trials.

#### 3.1.4. Budesonide

Budesonide is an anti-inflammatory agent which binds glucocorticoid receptors in bronchial cells, forming a complex that enters the nucleus to suppress transcription of pro-inflammatory genes and increase histone deacetylase 2 (HDAC2) activity, thereby reducing the production of cytokines such as interleukins and TNF [44]. It also promotes eosinophil apoptosis and inhibits activation of inflammatory cells, leading to decreased airway inflammation, bronchospasm, and hyperreactivity.

The distinct pharmacologic properties and mechanisms of these corticosteroids inform their selection and timing in neonatal care, with the shared goal of effectively reducing pulmonary inflammation and improving respiratory outcomes in preterm infants at risk of BPD. These steroids have varying modes of administration which also have important impacts on outcomes. However, routine early prophylaxis with budesonide is currently not advised.

### 3.2. Mode of Delivery

#### 3.2.1. Systemic (Intravenous or Oral) Route

The most well documented method of administration of postnatal corticosteroids to neonates is systemically by either intravenous infusion (IV) or orally (PO). There is also much evidence supporting both early and late systemic steroids’ efficacy in reducing BPD and the combined outcome of death or BPD [1,4,77,78]. These results are described in further detail below in the section reviewing the current status of systemic postnatal corticosteroid use for BPD.

#### 3.2.2. Inhaled Route

On the other hand, there is mounting evidence supporting the feasibility of inhaled or intratracheal corticosteroid use postnatally for infants with developing BPD. The inhaled route allows for absorption of the steroid directly into the airways via metered dose inhalers or jet nebulizers [79], whereas intratracheal administration uses surfactant as a vehicle to bypass the vascular supply and deliver medication directly to the lung parenchyma. In theory, the inhaled route could offer a more targeted approach potentially minimizing systemic adverse effects of corticosteroids [5]; however, it has yet to be proven as more effective than the former [80]. Less common alternatives include vibrating mesh and ultrasonic methods. While jet nebulizers allow flexible dosing, they are often limited by cost and treatment time [81]. Conversely, MDIs (using spacers and masks in newborns) offer faster, more consistent delivery [82,83]. In newborns, spacers and masks are used to improve delivery and minimize drug loss, and unlike jet nebulizers, MDIs provide only intermittent dosing but with shorter treatment times [79]. Neonatal lung models and radiolabeled studies demonstrate that MDIs deliver medication more efficiently than jet nebulizers, particularly during mechanical ventilation, though overall lung deposition remains remarkably low (<2%) [84,85,86,87,88,89,90,91,92,93,94,95,96]. Additionally, the placement of the device (e.g., directly at the endotracheal tube) and timing (number of breaths after actuation) significantly affect how much of the drug reaches the lungs. More specifically, in the case of corticosteroids, most trials have administered inhaled postnatal steroids (mainly budesonide) via MDI, with the exception of two studies published in the early 2000s [97,98].

A 2017 Cochrane review revealed no evidence supporting inhaled steroids over systemic steroids in the management of ventilator-dependent preterm infants [99]. Additionally, an open-label study of corticosteroid treatment (OSECT) compared early (<72 h) versus delayed treatment (>15 days) and systemic dexamethasone versus inhaled budesonide [100]. The results showed no significant differences between groups in the primary outcome of death or oxygen need at 36 weeks. The largest trial evaluating early inhaled budesonide compared to placebo, the Neonatal European Study of Inhaled Steroids (NEuroSIS) trial [61], randomized 863 infants born between 23 and 27 weeks’ gestation receiving respiratory support at <12 h of age to receive inhaled budesonide or placebo. Although inhaled budesonide was associated with a lower BPD rate, a small but non-significant increase in mortality was noted, arguing against routine clinical use of inhaled budesonide. No plausible explanation has yet been identified for this higher mortality, and two meta-analyses [101,102] did not find this association between inhaled postnatal corticosteroids and mortality. Finally, a 2016 systematic review found inconclusive evidence supporting that inhaled corticosteroids improve long-term outcomes in BPD [103]. On the other hand, a 2022 Cochrane review found that inhaled corticosteroids initiated late (≥ seven days of life) in preterm infants at risk of developing BPD reduces mortality or BPD at 36 weeks PMA [104].

#### 3.2.3. Intratracheal Route

Intratracheal delivery of postnatal corticosteroids using surfactant as a vehicle has limited data supporting it as an effective strategy for the prevention of BPD. Two RCTs conducted by Yeh et al. (a pilot study followed by a multicenter trial) [62,63] comparing intratracheal administration of surfactant/budesonide with that of surfactant alone on the incidence of death or BPD described that the surfactant/budesonide group had significantly decreased incidence of BPD or death without immediate adverse effects. This finding is supported by two meta-analyses comparing intratracheal surfactant/budesonide to surfactant alone with reduced odds of BPD at 36 weeks, death or BPD at 36 weeks, and significantly increased survival without BPD at 36 weeks in the surfactant/budesonide group [105,106]. However, the Preventing Lung disease Using Surfactant and Steroid trial (PLUSS), an international RCT across 21 neonatal intensive care units (NICUs) in 4 countries (14 in Australia, 5 in New Zealand, and 1 each in Singapore and Canada) included 1059 extremely preterm infants and published their findings in November 2024; no clear difference in survival free of BPD was noted between infants who received intratracheal budesonide mixed with surfactant and those who received surfactant only [64]. Taken together, while intratracheal budesonide mixed with surfactant did show promise in earlier studies and meta-analyses, the most recent large-scale trial does not confirm a clear benefit, highlighting the need for further high-quality evidence before this approach can be recommended for routine prevention of BPD. Additionally, the BIB Trial (Budesonide in Babies) [65], a large multicenter RCT, enrolled 641 infants (55.3% of planned *n* = 1160) and confirmed that combining budesonide with surfactant did not reduce the incidence of BPD or death at 36 weeks PMA in extremely preterm infants.

While approximate dose equivalencies between corticosteroids are often cited to guide clinical decisions (i.e., dexamethasone being about 25 times more potent than hydrocortisone), these conversions should be interpreted with caution. Potency estimates do not fully capture the differences in pharmacokinetics, receptor affinity, genomic and non-genomic effects, and risk profiles across agents. For reference, the cumulative exposure in key trials varied substantially: the STOP-BPD trial [50] administered a total hydrocortisone dose of 72.5 mg/kg over 22 days, the NICHD Hydrocortisone (HC) trial [51] used a low-dose hydrocortisone regimen totaling 7.5 mg/kg over 12 days, and the DART trial [53] employed a cumulative dexamethasone dose of 0.89 mg/kg over 10 days. These differences underscore that even when adjusted for relative potency, regimens are not directly interchangeable and may lead to distinct clinical outcomes.

## 4. Current Status of the Use of Systemic Postnatal Corticosteroids for BPD

In 1972, Liggins et al. first demonstrated that antenatal glucocorticoids accelerate fetal lung maturation, thereby reducing respiratory distress syndrome (RDS) and mortality in preterm infants [107]. Subsequent studies confirmed that antenatal steroid administration significantly lowers mortality, RDS, and intraventricular hemorrhage rates. In the early 1980s, high-dose dexamethasone was used to treat ventilator-dependent infants with BPD [108,109]. The results showed improved respiratory function and earlier extubation rates as a result of dexamethasone with few adverse effects. Following this discovery, postnatal corticosteroid use swiftly took over neonatal care in the early 1990s, climbing by 41% among infants born at less than 750 g by 1995 [110]. However, in 1998 a large multicenter follow-up study from Taiwan first diminished clinical confidence in postnatal steroids by revealing both an increase in adverse effects on neuromotor function and a doubling of the risk of cerebral palsy at 2 years of age in infants treated with a 4-week course of dexamethasone starting in the first 12 h of life [111]. Other studies promptly followed supporting these conclusions through the early 2000s, leading to the release of a statement by the American Academy of Pediatrics (AAP) in 2002 advising against using postnatal steroids to prevent or treat BPD in preterm infants due to concerns about cerebral palsy and adverse neurodevelopmental outcomes [112]. Over the years following, rates of postnatal steroid use decreased. The landmark DART study (Dexamethasone: A Randomized Trial) by Doyle et al. in 2006 was the first to document that low-dose dexamethasone administered after the first week of life in chronically ventilator-dependent infants facilitated MV weaning and extubation and improved lung function without the complications associated with higher doses [53,113]. The study enrolled 70 infants born at less than 28 weeks GA or under 1000 g (far from the target of 814 infants to be recruited), all of whom were ventilator-dependent. The participants were randomly assigned to receive dexamethasone (a cumulative dose of 0.89 mg/kg over 10 days) or a placebo. Infants in the dexamethasone group had a significantly higher extubation success rate compared to the placebo group (60% vs. 12%), and their follow-up study at 2 years of corrected gestational age revealed that there was no increase in short-term complications or neurodevelopmental impairments/death. Notably, no obvious adverse effects were observed regarding glucose levels, hypertension, or intestinal perforation. Various other studies have been published, with results attesting to the efficacy and safety of careful dexamethasone use [77,114,115,116]. Since this shift, researchers and practitioners have reintroduced postnatal steroid use with ongoing studies aiming to pinpoint an optimal dosing regimen and timing to balance the benefits and adverse effects.

Another randomized controlled trial evaluated the long-term outcomes of different dexamethasone tapering regimens in 59 extremely preterm infants (≤27 weeks’ gestation) with significant lung disease [71]. These patients remained ventilator-dependent with a mean airway pressure ≥ 8 cm H_2_O and FiO_2_ ≥ 0.6 between days 10 and 21 of life. Infants were randomized to receive either a 42-day slow taper (*n* = 30; cumulative dexamethasone dose: 7.56 mg/kg) or a 9-day rapid taper (*n* = 29; 4.04 mg/kg). The 42-day group had a shorter ventilation duration and higher intact survival at 7 years of age, defined as a normal neurologic examination, an intellectual quotient > 70, and independent school functions (75% vs 34%). Additionally, a cohort study analyzed 482 matched pairs of infants born at <27 weeks’ gestation from 45 US hospitals in the NICHD Neonatal Research Network (2011–2017), comparing systemic corticosteroid therapy (mostly dexamethasone (*n* = 363, 75.3%)) initiated between days 8 and 42 with no treatment to assess its impact on death or neurodevelopmental impairment at 2 years corrected age [78]. The results showed that corticosteroids reduced the risk of death or disability in infants with a high pretreatment risk of severe BPD or death but were associated with potential harm in the lower-risk infants. Corroborating these results, a meta-analysis of 23 randomized control trials evaluated exposure to two types of postnatal steroids (21 studies on dexamethasone and 2 on hydrocortisone) beyond the first week of life and described that they were associated with a decrease in mortality at 28 days; extubation failure by 3, 7 and 28 days post-exposure; and BPD at 36 weeks PMA, without significant differences in the composite outcome of death or cerebral palsy [117]. A 2024 systematic review evaluating hydrocortisone and dexamethasone in preterm infants found that neonatal dexamethasone offers greater respiratory benefits than hydrocortisone but warranted caution due to potential risks, while current evidence remains inconclusive regarding long-term adverse effects or neurodevelopmental consequences of systemic steroid use in this population [118]. In addition, a meta-regression analysis demonstrated that postnatal dexamethasone, but not hydrocortisone, when administered after the first week of life, was associated with a decrease in death or cerebral palsy when administered in infants at high risk of BPD (>50% rate of BPD in the control group) [4]. Trials on the use of postnatal hydrocortisone in ventilator-dependent premature infants after 1 week of life have not shown a similar effect towards improved BPD rates or mortality [50,51]. As discussed above, the results of the Watterberg et al. trial on hydrocortisone [51] showed that treatment did not differentially affect outcomes based on baseline risk for higher grades of BPD or death. Additionally, the STOP-BPD trial demonstrated that initiating hydrocortisone between days 7 and 14 did not improve the rate of survival without BPD compared to placebo [50]. Recent evidence suggests that the impact of corticosteroids on survival and long-term outcomes is highly dependent on the baseline risk of the infant. Specifically, corticosteroids have been shown to reduce the risk of death or disability in infants with a high pretreatment risk of severe BPD or death, but they may be associated with potential harm in lower-risk infants. Therefore, clinicians must utilize risk-stratified approaches rather than assuming a universal survival benefit.

**Figure 3:**
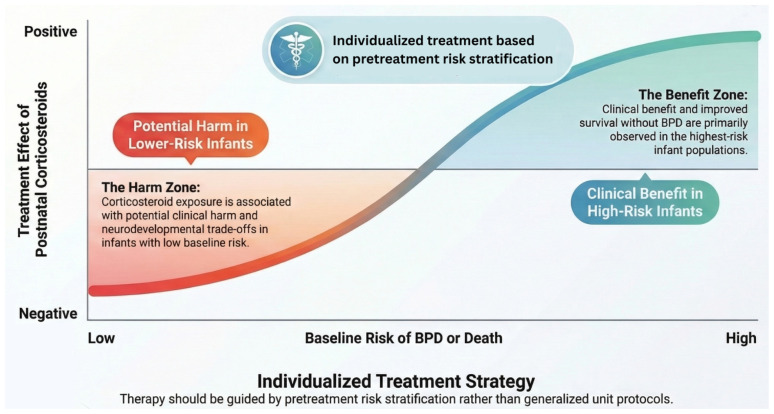
Conceptual graph of the postnatal corticosteroid treatment effect relative to baseline risk.

Although results regarding the selective use of postnatal corticosteroids beyond the first week of life are promising, their use remains tempered by concerns regarding potential adverse effects, particularly when administered during critical periods of organ maturation. However, these concerns regarding adverse neurodevelopmental outcomes are primarily observed with early high-dose regimens, and more recent data evaluating low-dose systemic postnatal corticosteroids after the first week of life in high-risk infants have not demonstrated a similar risk profile. Studies have reported short- and long-term adverse effects of steroids in newborns, including hyperglycemia, transient high systolic blood pressure and cerebral palsy [52,117]. Reports on cerebral palsy are isolated to the administration of high dosages of dexamethasone within the first week of postnatal life [52,117]. Animal primate studies have outlined that dexamethasone may affect the micro-structure of the brain [119,120]. On the other hand, a prospective cohort study including 392 preterm infants found that low-dose dexamethasone initiated after the first postnatal week for evolving BPD was associated with larger cerebellar and subcortical grey matter volumes at term-equivalent age, as well as improved motor scores at two years, suggesting potential neuroprotective benefits rather than harm in selected high-risk patients [121]. In addition, glucocorticoids have been associated with the arrest of alveolar epithelial cell growth, mediated by induction of the cell cycle inhibitor p21^CIP1^, which impairs normal lung development [122]. As with any therapy, the benefits must be weighed against the risks. The inflammatory burden from the postnatal environment and cumulative hypoxic injury in the immature brain may be more detrimental than the potential side effects of postnatal dexamethasone in the most high-risk infants, as outlined by the results of the Doyle et al. meta-regression reports [4,114]. This is in line with the trial comparing two dexamethasone regimens in high-risk infants and reporting that a higher dose administered after the first week of life was associated with improved survival and better neurodevelopmental outcomes at 7 years of age [71]. Crucially, the treatment effect is highly dependent on baseline risk; corticosteroids have been shown to reduce the risk of death or disability in infants with a high pretreatment risk of severe BPD or death, whereas they may be associated with potential harm in infants at lower baseline risk. Figure 3 illustrates this treatment effect relative to baseline risk conceptually.

The use of postnatal corticosteroids in preterm infants has risen over the past decade, yet dosing regimens, criteria for their use, and time of initiation all remain highly variable across jurisdictions. A large international cohort study of over 47,000 preterm infants from seven high-income countries [123] found that systemic postnatal corticosteroid use increased from 18% in 2010 to 26% in 2018, yet this rise was not accompanied by any reduction in BPD rates. Across 203 NICUs in the United Kingdom and Ireland, dexamethasone regimens varied with starting doses from as low as 0.05 to as high as 0.5 mg/kg/day [124], delivered both to ventilated infants as well as those receiving non-invasive respiratory support. Additionally, between 2012 and 2019 in England and Wales, around 1 in 12 infants born < 32 weeks and 1 in 4 born < 27 weeks received postnatal corticosteroids for pulmonary indications [125]. On the other hand, in another large Canadian cohort [126], most infants received systemic steroids between the third and fifth weeks of life, with the fourth week being the most common starting point. Observational evidence suggests that this timing may not be optimal [8] and that perhaps individualized treatment strategies tailored to each infant’s disease trajectory may ultimately prove more effective. This marked variability and lack of consensus highlight an urgent need to define clear recommendations for steroid selection, dosing regimens, and timing of initiation to improve both efficacy and safety outcomes for preterm infants at risk of BPD.

While researchers continue working towards establishing these guidelines, over the past few decades postnatal steroid use has shifted primarily to facilitating weaning infants from invasive respiratory support such as MV [127]. MV is an important potentially modifiable risk factor for the development of BPD [128]. Prolonged duration of ventilation not only reflects the severity of underlying lung immaturity in preterm neonates but also actively disrupts alveolarization and microvascular development through barotrauma, volutrauma, and oxygen toxicity [2,10,13]. In the extreme preterm population, 70–100% of infants born at 22–28 weeks of gestation are exposed to mechanical ventilation, with nearly 50% being ventilated for ≥3 weeks [129]. In Norway, babies born at 23 weeks’ gestation spent an average of 32 days on mechanical ventilation [130]. There is potential to lessen pulmonary simplification and improve long-term respiratory outcomes via judicious use of systemic corticosteroids to reduce the duration of ventilation and mitigate ventilator-associated injury [131].

The collective results described above, along with the associations of severe BPD and prolonged mechanical ventilation with poor long-term neurodevelopmental outcomes [77], prompted the Canadian Paediatric Society to recommend in 2020 that “for infants who remain ventilated after the first week post-birth with increasing oxygen requirements and worsening lung disease, the benefits of dexamethasone therapy appear to outweigh the potential adverse effects. In these circumstances, low-dose dexamethasone (with an initial dose of 0.15 mg/kg/day to 0.2 mg/ kg/day, tapered over a short course [7 to 10 days]) should be considered” [8]. As such, when the risk of death or BPD is high, the trade-off between beneficial and harmful effects may favor the use of low–medium-dose postnatal corticosteroids after the first week of postnatal life for improved long-term outcomes.

## 5. Non-Invasive Modalities for Monitoring Pulmonary Status Before/During and After Postnatal Corticosteroid Use

It is increasingly evident that defining the most appropriate cumulative dose, treatment duration, and tapering schedule of postnatal corticosteroids remains a major challenge, especially when attempting to balance anti-inflammatory efficacy against short- and long-term adverse effects. The traditional “one size fits all” approach may no longer be defensible in the face of accumulating evidence showing variable responses and toxicity profiles across individuals. To address this gap, future research should prioritize more sophisticated phenotyping strategies to individualize therapy regimens that are being studied in trials and according to each patient’s inflammatory burden, disease trajectory, and comorbidities. One promising avenue is the use of lung ultrasound as a bedside tool to monitor dynamic changes in lung aeration, quantify pulmonary edema, and track the evolution of lung scores in response to corticosteroid treatment. Additionally, serial echocardiographic assessments could help clinicians detect early evidence of cardiac side effects, such as left ventricular hypertrophy or impaired diastolic function, as well as provide real-time information on the status of the PDA, which may be influenced by steroid exposure. The ongoing Surveillance of Postnatal steroids Effects on Cardiac function in extremely preterm infants with evolving lung disease (SPEC) study is prospectively evaluating many of these points in a cohort of extremely preterm infants undergoing PNS treatment for severe respiratory disease (ClinicalTrials.gov ID NCT04644094). There is a possibility that some infants may demonstrate a paradoxical response to steroids, in which the reduction of pulmonary inflammation leads to a decline in pulmonary vascular resistance. If the duct remains patent and large, this drops in resistance could increase transductal left-to-right shunting, further exacerbating pulmonary edema and compromising respiratory function. This heterogeneity in physiological response between patients underscores the need to recognize that not all patients will react uniformly to treatment. Incorporating adjunctive monitoring tools into clinical practice and research, first through observational mechanistic studies and ultimately within RCTs, may help tailor interventions more precisely. Together, the emerging imaging modalities described below could enable a more tailored and responsive approach to postnatal corticosteroid administration, improving both safety and treatment effectiveness in preterm infants at risk of bronchopulmonary dysplasia.

### 5.1. Lung Ultrasound

**Figure 4:**
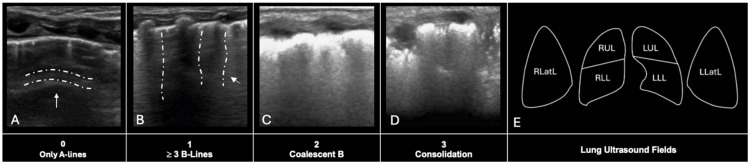
Lung ultrasound scoring at the Montreal Children’s Hospital NICU. (**A**) A score of 0, characterized by a clear pleural line and the presence of horizontal A-lines (indicated by the arrow) depicting a well-aerated lung; (**B**) A score of 1, showing at least three well-spaced vertical B-lines (indicated by the arrow); (**C**) A score of 2, representing confluent/coalescent B-lines; (**D**) A score of 3, indicating extensive lung consolidation; (**E**) 6-zones scanned in our protocol, the three sections scanned per lung (typically the upper anterior, lower anterior, and lateral zones) to calculate the total lung ultrasound score.

Lung ultrasound (LUS) is a tool that can estimate underlying lung aeration, giving an indirect assessment and appreciation of pulmonary interstitial fluid and collapse [132]. Additionally, it has been increasingly used in preterm infants to evaluate several pathologies [133], for decisional algorithms of surfactant administration [134,135], and to predict moderate–severe BPD at 4 weeks of age [136]. Pulmonary edema, atelectasis, and fluid accumulation in the pulmonary interstitium can lead to increased LUS estimates of pulmonary densification, as they enhance the presence of ultrasonographic “B-lines”, subpleural consolidations, and reduced lung aeration, reflecting underlying lung congestion and impaired gas exchange [137]. Since the emergence of lung ultrasound as a non-invasive tool for assessing neonatal lung aeration, a number of scoring systems have been proposed, Brat’s [134], Raimondi’s [138] and Rodriguez-Fanjul’s [138] being the three main systems. Research comparing their utility (specifically in the context of predicting surfactant need) has suggested that they each confer an ability to predict the need for surfactant use [139]. The authors recommend that the same system be used consistently within the same center; as such, the Brat system is described in Figure 4.

Studies have described LUS as a tool for estimating lung aeration and tracking the evolution of BPD in preterm infants [140,141,142,143]. These studies have explored its predictive value, role in guiding therapeutic interventions, and potential for early diagnosis and monitoring. Using the NICHD 2001 BPD classification, a prospective study [144] enrolled 87 infants, categorizing them into mild (*n* = 39), moderate (*n* = 33), and severe (*n* = 15) BPD. The global whole-chest LUS score correlated with BPD severity and showed a progressive improvement over time, approaching normalization by discharge. In a cohort of 62 preterm infants, LUS scores gradually decreased from birth through early infancy [145]. Although also decreasing in time, those who developed BPD exhibited consistently higher LUS scores. A prospective cohort study of 108 preterm infants [146] (<37 weeks GA) assessing LUS features in BPD found that infants with moderate-to-severe BPD had significantly thicker and rougher pleural lines, diffuse alveolar–interstitial syndrome, retrodiaphragmatic hyperechogenicity, small cysts, and rough diaphragmatic morphology. These findings highlight LUS as a radiation-free tool for assessing BPD severity, enabling early risk stratification and guiding clinical decisions. Building on this, Liu et al. evaluated 130 very low-birth-weight (VLBW) infants (<32 weeks GA) to determine LUS’s predictive accuracy for BPD [147]. Using 12-, 10-, and 6-region scoring protocols, LUS was performed on postnatal days 1–15. The 12- and 10-region protocols reliably predicted BPD from days 9 to 15, whereas the 6-region protocol had lower predictive accuracy on days 6 and 9. Additionally, integrating clinical variables such as GA and MV improved predictive performance. These findings suggest that LUS could be an emerging early predictor of BPD. Beyond prediction, LUS has been explored as a tool for monitoring treatment response. Alonso-Ojembarrena et al. assessed 18 preterm infants [136] (<32 weeks GA) to determine whether LUS scores change with diuretic therapy. Infants were categorized as responders or non-responders based on respiratory support (RS) evolution. Responders had a shorter MV duration, lower rates of moderate-to-severe BPD, and significantly decreased LUS scores within 2 days post-diuretics, with continued improvement at 1 and 3 weeks. Extubation rates were also higher among responders. These findings suggest that LUS can track diuretic response, with improvements aligning with RS weaning, potentially guiding individualized BPD management. Similarly, Radulova et al. prospectively studied 124 VLBW infants to evaluate LUS as a predictor of BPD [148]. Infants were stratified by GA (<28 weeks vs. 28–32 weeks) and BPD severity. Weekly LUS was performed until 36 weeks PMA, with LUS scores calculated on day 7 based on aeration in six lung regions. BPD infants had significantly higher LUS scores (>8 on day 7, *p* < 0.001) and more lung consolidations compared to non-BPD infants (median: 14 vs. 2.5, *p* < 0.001), particularly between 1 week and 1 month (*p* = 0.001). In <28-week infants, lung consolidations appeared across all lung fields, whereas in 28–32-week infants, lung consolidations were more common in anterior–lateral fields. These findings indicate that LUS score > 8 on day 7 and widespread lung consolidations are early predictors of moderate-to-severe BPD, supporting LUS as a non-invasive monitoring tool in preterm infants. LUS has also been shown to be a valuable tool for assessing lung aeration in animal models. Loi et al. investigated a hyperoxia-exposed preterm rabbit model to determine its similarity to evolving BPD in human neonates [143]. The study included 25 preterm rabbits (28 days gestation) and 25 neonates (mean GA: ~26 weeks), comparing lung aeration, LUS findings, and lung mechanics. Both rabbits and neonates exhibited heterogeneous lung aeration abnormalities consistent with evolving BPD. Interrater agreement for LUS scoring in rabbits was high (intra-class coefficient: 0.989, *p* < 0.0001), and lung mechanics were similarly altered in both groups. LUS scores also correlated with airway resistance in both rabbits (ρ = 0.519, *p* = 0.008) and neonates (ρ = 0.409, *p* = 0.042). Additionally, 13 term rabbits under normoxia had no ultrasound or histological abnormalities, confirming that hyperoxia exposure drives the observed changes. This study outlines the preterm rabbit as a model for evolving BPD and supports LUS as a valuable tool for both research and clinical applications. In addition, the Serial Lung Ultrasound in predicting the need for surfactant and Respiratory course in Preterm infants (SLURP) study [149]—whose aim was to evaluate the learning curve and effectiveness of structured training in LUS among novice operators across three NICUs in the United Kingdom—described LUS as an effective diagnostic tool when performed in the first 3 h of life for predicting a need for surfactant in preterm infants. Beyond its predictive value, LUS might serve as a physiology-driven guide for corticosteroid initiation by identifying infants with high LUS scores reflecting severe pulmonary densification and interstitial edema. While evidence from other therapies, such as diuretics, suggests that falling LUS scores correlate with clinical improvement, the direct correlation between a reduction in LUS scores and successful steroid-induced respiratory recovery remains a hypothesis. This relationship is currently being evaluated in prospective studies, such as the NORDIC-SPEC study (Neonatal Outcomes Related to the early Discovery of Impaired Cardiac Function: Surveillance of Postnatal steroids Effects on Cardiac function in extremely preterm infants with evolving lung disease—ClinicalTrials.gov ID: NCT04644094) to determine if real-time changes in lung aeration can reliably inform the optimal timing and monitoring of postnatal steroid therapy.

### 5.2. Respiratory Oscillometry and Respiratory System Reactance (Xrs)

An additional bedside tool that has been explored for its capacity to assess airway resistance and lung compliance is respiratory oscillometry [150,151,152,153]. More specifically, respiratory system reactance (Xrs) has been shown to be sensitive particularly to peripheral airway obstruction and alveolar recruitment [154,155,156,157]. Oscillometry is used to analyze pressure and flow signals generated during spontaneous breathing [158]. Recent multicenter evidence shows that measuring Xrs on the seventh day of life significantly improves prediction of respiratory outcomes, including BPD and duration of respiratory support, beyond established clinical models (NICHD) and gestational age or birth weight alone [159].

These findings collectively reinforce both LUS and Xrs as non-invasive tools for assessing lung aeration, predicting BPD severity, and monitoring disease progression, with growing evidence supporting their integration into both clinical management and research. In future studies, markers detecting a decrease in pulmonary and systemic inflammation may allow for tailoring of corticosteroid therapy. Identifying markers that reflect a patient’s real-time lung status and response to steroids, such as changes in LUS scores or Xrs, may help clinicians individualize and optimize systemic postnatal corticosteroid therapy, balancing efficacy and safety in the management of evolving BPD.

### 5.3. Electrical Impedance Tomography (EIT)

Electrical impedance tomography (EIT) offers a non-invasive, real-time method using surface electrodes to generate bedside maps of regional lung ventilation and aeration [160]. EIT has been best described in its use for early prediction of respiratory outcomes, assessment of ventilation distribution, and optimization of ventilator settings in preterm infants [161]. As such, although it has not yet been used for this purpose, EIT may be particularly valuable for tracking responses to systemic corticosteroids in preterm infants with evolving BPD. Corticosteroids are expected to improve lung aeration by reducing inflammation and edema changes that EIT can detect through increased aerated lung volume and improved homogeneity. Gaertner et al. [162] demonstrated that early EIT markers predicted oxygen need at 28 days, suggesting potential for EIT to capture treatment effects before clinical signs evolve. Furthermore, Brown et al. [163] highlighted EIT’s sensitivity to maturational changes in lung structure, supporting its role in assessing corticosteroid-induced lung development. EIT could thus help stratify responders and guide individualized therapy in real time.

### 5.4. Static Pulmonary Imaging

Static pulmonary imaging, including chest radiography (CXR), computed tomography (CT), and magnetic resonance imaging (MRI), complements bedside monitoring by providing detailed structural assessments. While CXR remains the clinical routine for assessing immediate structural changes, advanced modalities like CT and MRI are primarily research-focused tools in the neonatal population [164]. CT offers high anatomical resolution but is limited by radiation concerns, whereas lung MRI provides a radiation-free assessment of parenchyma and perfusion [165]. Although technically challenging in neonates, lung MRI is gaining importance in longitudinal research and phenotyping of chronic lung disease [166], as exemplified by the ongoing EMBLEM study (Early-life MRI Biomarkers of Longer-term Respiratory Morbidity in Infants Born Extremely Preterm) (ClinicalTrials.gov ID: NCT06065215) investigating biomarkers for long-term morbidity. A summary of concomitant monitoring tools for postnatal corticosteroids’ effects on the respiratory system in preterm infants with evolving BPD can be found in Table 4.

### 5.5. A Physiology-Driven Decision-Making Framework

To improve bedside applicability, we propose a clinical algorithm that shifts the focus from empiric protocols toward precision-based care. The framework begins with risk stratification based on the infant’s gestational age and current level of respiratory support.

- Phase 1: Identification of High-Risk Candidates. Infants remaining on invasive mechanical ventilation after the first postnatal week with increasing oxygen requirements should be prioritized for evaluation.
- Phase 2: Physiological Assessment. Clinicians should utilize non-invasive tools, such as LUS, to confirm a high inflammatory/edema burden. High airway resistance as measured by respiratory oscillometry may further support the need for intervention.
- Phase 3: Regimen Selection. Based on the assessment, the framework suggests low-dose dexamethasone (DART regimen) for infants difficult to extubate after day 7, or hydrocortisone for those with evolving disease earlier in the clinical course.
- Phase 4: Monitoring for Response and Harm. Following initiation, serial monitoring with LUS and bedside echocardiography (for cardiac side effects) ensures that the treatment is both effective and safe.

## 6. Conclusions

Systemic postnatal corticosteroids remain a pivotal intervention for survival and in the management of evolving bronchopulmonary dysplasia in extremely preterm infants. While their anti-inflammatory properties facilitate weaning of MV and extubation and reduce pulmonary morbidity, their broader physiologic effects—particularly on the immature lung—are incompletely understood. Current clinical practices lack standardization in timing, dosing, and monitoring, often relying on generalized protocols rather than individualized physiologic markers. However, the emergence of advanced, non-invasive monitoring tools—such as lung ultrasound, respiratory oscillometry, and electrical impedance tomography—offers new opportunities to assess fluid content, lung compliance and lung aeration in real time. These modalities may enable more nuanced assessments of steroid responsiveness and pulmonary trajectory, ultimately guiding safer and more effective use of corticosteroids. Moreover, the development of clinical protocols will allow consistency in their use for specific clinical situations, promoting more individualized care and avoiding unnecessary variations in practice. Moving forward, integrating these tools into both research and clinical practice will be essential for transitioning from empiric to precision-based neonatal respiratory care.

## 7. Directions for Future Research

Despite growing recognition of the systemic effects of postnatal corticosteroids in preterm infants, many knowledge gaps persist regarding their precise pulmonary, cardiovascular, and autonomic impacts. To advance the field toward individualized corticosteroid therapy, future research must adopt a multisystem, longitudinal approach that captures the dynamic interplay between pulmonary, cardiovascular, and autonomic physiology in preterm infants. Future investigations should:

- Characterize the temporal effects of corticosteroids on lung structure and function, using serial assessments with lung ultrasound, oscillometry, and electrical impedance tomography.
- Define normative trajectories and thresholds for these physiologic markers in steroid-treated versus untreated infants.
- Correlate pulmonary changes with systemic responses, including cardiac remodeling, ductal dynamics, and autonomic regulation, to identify physiologic phenotypes of steroid responders and non-responders.
- Develop predictive models that integrate multimodal data to guide real-time steroid decision-making.

This research agenda will not only refine respiratory management but also lay the groundwork for a companion investigation into the cardiovascular consequences of systemic corticosteroids. Part two of this series will explore how PNS exposure influences cardiac function, and hemodynamics—particularly in the context of evolving lung disease cardiac hypertrophy and ductal physiology. By bridging pulmonary and cardiovascular domains, this integrated framework aims to support precision neonatal corticosteroid therapy tailored to the unique developmental trajectory of each infant.

The NORDIC-SPEC study (Neonatal Outcomes Related to the early Discovery of Impaired Cardiac Function: Surveillance of Postnatal steroids Effects on Cardiac function in extremely preterm infants with evolving lung disease—ClinicalTrials.gov ID: NCT04644094) represents one such effort, aiming to characterize the cardiopulmonary effects of dexamethasone in extremely preterm infants treated for evolving lung disease after the first postnatal week. This prospective observational study will assess changes in cardiac morphology and function, ductal patency, lung ultrasound scores, and heart rate variability before, throughout and after postnatal steroidal exposure to corticosteroids. By evaluating how dexamethasone modifies these physiologic markers and correlating them with clinical outcomes, SPEC seeks to identify subgroups of infants who are most likely to benefit, or be harmed, by treatment. Hypotheses include the occurrence of early, transient left ventricular hypertrophy, a steroid-induced reduction in pulmonary inflammation evidenced by falling LUS scores, and enhanced autonomic regulation reflected by improved HRV. Beyond individual studies, future research must also define normative and pathological trajectories for these novel metrics, determine their predictive validity, and explore their utility in guiding real-time steroid decision-making. Ultimately, integrating multimodal physiologic monitoring into clinical algorithms may support more personalized, targeted steroid use, shifting the field from generalized protocols toward precision neonatal care.

## Figures and Tables

**Table 1:** Existing definitions of BPD based on physiological oxygen-based criteria compared with respiratory support-based grading systems. The population is premature infants (<32 weeks gestational age), and the time of BPD severity assessment is 36 weeks PMA or discharge home, whichever comes first. Abbreviations: PMA (post-menstrual age); RA (room air); PPV (positive pressure ventilation); NCPAP (nasal continuous positive pressure airway); IPPV (intermittent positive pressure ventilation); NIPPV (non-invasive positive pressure ventilation); LFNC (low-flow nasal cannula); HFNC (high-flow nasal cannula); SIPAP (synchronized inspiratory positive airway pressure); NIHFV (non-invasive high-flow ventilation); MV (mechanical ventilation); CNN (Canadian Neonatal Network).

Definition	Nil	Grade 1 or Mild	Grade 2 or Moderate	Grade 3 or Severe
Jobe (2001) [13]	O_2_ duration for <28 days after birth	On RA	Need for <30% O_2_	Need for ≥30% O_2_ and/or positive pressure (PPV or NCPAP)
Walsh (2003, 2004) [22,23]	On RA with an O_2_ saturation ≥ 88%, or passing a timed, continuously monitored oxygen reduction test	On MV, CPAP or with supplemental O_2_ > 0.30 without additional testing - If supplemental O_2_ < 0.30, infant undergoes oxygen reduction test: saturation < 88% (80 to 87% for 5 minutes, or <80% for 1 min) - If supplemental O_2_ <0.30, infant undergoes oxygen reduction test: saturation < 88% (80 to 87% for 5 minutes, or <80% for 1 min)		
Abman (2017) [17]	O_2_ treatment for <28 days and breathing RA	O_2_ treatment ≥ 28 days and breathing RA	O_2_ treatment ≥ 28 days and receiving < 30% O_2_	Type 1: O_2_ treatment ≥ 28 days and ≥30% O_2_ or NCPAP/HFNC; Type 2: O_2_ treatment ≥ 28 days and MV
Higgins (2018) [14]	No assessment of O_2_ duration and no oxygen or other respiratory support at 36 weeks PMA	No invasive IPPV; or NCPAP, NIPPV or LFNC ≥ 3 L/min, FiO_2_ 21; or LFNC 1- < 3 L/min, FiO_2_ 22–29; or hood FiO_2_ 22–29; or LFNC < 1 L/min, FiO_2_ 22–70	Invasive IPPV, FiO_2_ 21; or NCPAP, NIPPV or LFNC ≥ 3 L/min, FiO_2_ 21; or LFNC 1- < 3 L/min, FiO_2_ ≥30; or hood FiO_2_ of ≥30; or LFNC < 1 L/min, FiO_2_ > 70	Invasive IPPV, FiO_2_ at >21; or NCPAP, NIPPV or LFNC ≥ 3 L/min, FiO_2_ at ≥30
Jensen (2019) [16]	No assessment of O_2_ duration, and no O_2_ or other respiratory support at 36 weeks PMA	LFNC ≤ 2 L/min regardless of FiO_2_ and no other respiratory support	NCPAP, NIPPV, or LFNC >2 L/min regardless of FiO_2_	Invasive PPV regardless of FiO_2_
CNN (2018) [24]	No respiratory support, and if there is it is for an acute event (the infant prior to this event was on RA for a prolonged period)	Headbox or incubator, FiO_2_ > 21; or LFNC < 0.1 L/min, FiO_2_ 100; or LFNC < 1.5 L/min, FiO_2_ 21–99	LFNC ≥ 0.1 L/min, FiO_2_ 100; or LFNC ≥ 1.5 L/min, FiO_2_ ≥ 21–29; or CPAP, SIPAP, NIPPV, NIHFV, FiO_2_ 21–29	LFNC ≥ 1.5 L/min, FiO_2_ ≥ 30; or CPAP, SIPAP, NIPPV, NIHFV, FiO_2_ ≥ 30; or MV (intubated), FiO_2_ ≥ 21–100

**Table 2:** Pharmacological properties and relative potency of postnatal corticosteroids used in BPD [46,47,48].

Mode of Delivery	Steroid	Mechanism of Action			
		Equivalent Dose (mg)	Potency		Half-Life (h)
			GC	MC	
Systemic (IV or PO)	HC	20	1	1	8–12
	Dexa	0.75	25	0	36–54
	Beta	0.75	25	0	36–54
	Prednisone/olone	5	4	0.8	12–36
	Methylprednisone/olone	4	5	0.5	12–36
Inhaled	Budesonide	0.375	9	~0	2–3
Intratracheal	Budesonide + Surfactant				

Additional corticosteroids sometimes used in same/similar contexts (such as beclomethasone diproprionate, fluticasone, flunisolide, and fluticasone propionate) have not been included here as they remain under-studied. Abbreviations: HC (hydrocortisone); Dexa (dexamethasone); Beta (betamethasone); GC (glucocorticoid); MC (mineralocorticoid); IV (Intravenous); PO (Oral).

**Table 3:** Characteristics, primary clinical roles, and level of evidence for postnatal corticosteroids in infants with evolving BPD.

Mode of Delivery	Steroid	Evidence in Preterm Infants with Evolving BPD	Primary Clinical Role	Level of Evidence
Systemic (IV or PO)	HC (early * low dose)	PREMILOC: Increased survival without BPD; potential risk of GI perforation when combined with NSAIDs or sepsis [49].	Early prophylaxis and BPD prevention	Moderate (multiple RCTs, e.g., PREMILOC)
	HC (high dose)	STOP-BPD: No improvement in survival without BPD (when administered between days 7 and 14) [50]. NICHD HC Trial: Not substantially higher survival without modest/severe BPD (when administered between days 14 and 28) [51].	Treatment of established BPD in ventilated infants	Moderate (RCTs with negative or mixed results, e.g., STOP-BPD)
	Dexa (early high dose)	Established efficacy in reducing BPD; associated with increased risk of GI perforation and cerebral palsy in early studies [52].	Facilitation of extubation and weaning from mechanical ventilation	High (strong RCT evidence of efficacy but also significant harm)
	Dexa (late low dose)	DART: Underpowered trial given the small sample size. Facilitates extubation; reduces time spent on ventilation, especially in very preterm, very low-birth-weight infants; less concern about neurodevelopment compared to early high-dose regimens [53].	Historical BPD prevention (no longer recommended due to safety)	High (strong evidence from RCTs and meta-analyses, e.g., DART)
	Beta	Primarily used antenatally for fetal lung maturation [54]; limited data on postnatal use; associations with PDA closure [55].	Antenatal fetal lung maturation (primary); potential postnatal role in facilitating PDA closure	Low (limited postnatal data; primarily observational)
	Prednis-one/olone	Limited neonatal BPD data (no prospective RCT has been performed evaluating its effectiveness in treating BPD); demonstrated association with weaning respiratory support and supplemental oxygen when given after 36 weeks PMA [56,57].	Management of refractory BPD and late weaning (often >36 weeks PMA)	Low (primarily observational data/case series; no prospective RCTs)
	Methyl- prednis one/olone	Limited evidence; preliminary results have shown associations with successful weaning of respiratory support [58,59] but also with no improvement in pulmonary severity scores and high numbers of infections [60].		
Inhaled	Budesonide	NEuroSIS trial: Reduced BPD incidence but with increased mortality; not recommended for routine use [61].	Early BPD prevention (routine use currently not advised)	Moderate (large RCTs, e.g., NEuroSIS; safety concerns remain)
Intratracheal	Budesonide + surfactant	Yeh et al.: Reduced BPD/death in pilot/multicenter RCTs without immediate adverse effects [62,63]. PLUSS: No clear benefit in survival without BPD [64]. BiB Trial: No reduced risk of BPD or death at 36 weeks PMA [65].	Prevention of BPD in extremely preterm infants	Moderate (mixed results from large RCTs, e.g., Yeh et al. vs. PLUSS/BiB)

Abbreviations: HC (hydrocortisone); Dexa (dexamethasone); Beta (betamethasone); PREMILOC (Trial to Prevent Bronchopulmonary Dysplasia in Very Preterm Neonates); GI (gastrointestinal); NSAIDs (non-steroidal anti-inflammatory drugs); NEuroSIS (Neonatal European Study of Inhaled Steroids); PLUSS (Intratracheal Budesonide Mixed With Surfactant for Extremely Preterm Infants: The PLUSS Randomized Trial); IV (Intravenous); PO (Oral); DART (Dexamethasone: A Randomized Trial). * Early exposure refers to postnatal corticosteroid exposure within the first 7 days of life and late refers to exposure after the first 7 days of life.

**Table 4:** Summary table of concomitant monitoring tools for postnatal corticosteroids’ effects on the respiratory system in preterm infants with evolving BPD.

Monitoring Tool	Clinical Parameters Assessed	Strengths	Limitations	Potential Role in Steroid Decision-Making
Lung Ultrasound (LUS)	Lung aeration, interstitial fluid, and BPD severity	Bedside, radiation-free; validated scoring	Operator-dependent; no universal scoring standard	Guides timing of initiation based on lung fluid/densification; monitors real-time recovery
Respiratory Oscillometry and Respiratory System Reactance (Xrs)	Airway resistance and lung compliance	Bedside; predictive of BPD risk by day 7	Requires specialized equipment and expertise	Early risk prediction to identify candidates for steroids; monitors airway changes
Electrical Impedance Tomography (EIT)	Regional ventilation and aeration maps	Real-time; tracks ventilation distribution	Primarily research use; requires expertise	Tracks acute response to therapy via ventilation homogeneity; stratifies responders
Static Pulmonary Imaging (CXR, CT, MRI)	Structural detail and BPD phenotype	High resolution; widely available (CXR)	Radiation (CT); technically demanding (MRI)	Long-term phenotyping; primarily for research and longitudinal follow-up

Abbreviations: BPD (bronchopulmonary dysplasia); CXR (chest X-ray); CT (computed tomography); MRI (magnetic resonance imaging); EIT (electrical impedance tomography); LUS (lung ultrasound); Xrs (respiratory system reactance), RDS (respiratory distress syndrome).

## Data Availability

No new data were created.

## Abbreviations

The following abbreviations are used in this manuscript:

PMA	Post-menstrual age
RA	Room air
PPV	Positive pressure ventilation
NCPAP	Nasal continuous positive airway pressure
IPPV	Intermittent positive pressure ventilation
NIPPV	Non-invasive positive pressure ventilation
NC	Nasal cannula
HFNC	High-flow nasal cannula
SIPAP	Synchronized inspiratory positive airway pressure
NIHFV	Non-invasive high-flow ventilation
MV	Mechanical ventilation
HC	Hydrocortisone
Dexa	Dexamethasone
Beta	Betamethasone
GC	Glucocorticoid
MC	Mineralocorticoid
PREMILOC	Trial to Prevent Bronchopulmonary Dysplasia in Very Preterm Neonates
NSAIDs	Non-steroidal anti-inflammatory drugs
NDI	Neurodevelopmental impairment
NEuroSIS	Neonatal European Study of Inhaled Steroids
PLUSS	Intratracheal Budesonide Mixed with Surfactant for Extremely Preterm Infants: The PLUSS Randomized Trial
IV	Intravenous
PO	Oral
